# Clinical and molecular landscape of metastatic extramammary Paget’s disease

**DOI:** 10.1093/oncolo/oyaf235

**Published:** 2025-07-28

**Authors:** Satomi Watanabe, Koji Haratani, Tomohiro Nakayama, Takayuki Takahama, Tsutomu Iwasa, Masayuki Takeda, Hidetoshi Hayashi

**Affiliations:** Department of Medical Oncology, Kindai University Faculty of Medicine, Osaka-Sayama 589-8511, Japan; Department of Medical Oncology, Kindai University Faculty of Medicine, Osaka-Sayama 589-8511, Japan; Department of Medical Oncology, Dana-Farber Cancer Institute, Boston, MA 02215, United States; Department of Medical Oncology, Kindai University Faculty of Medicine, Osaka-Sayama 589-8511, Japan; Department of Medical Oncology, Kindai University Faculty of Medicine, Osaka-Sayama 589-8511, Japan; Genome Medical Center, Kindai University Hospital, Osaka-Sayama 589-8511, Japan; Department of Medical Oncology, Kindai University Faculty of Medicine, Osaka-Sayama 589-8511, Japan; Department of Medical Oncology, Kindai University Faculty of Medicine, Osaka-Sayama 589-8511, Japan; Department of Medical Oncology, Kindai University Faculty of Medicine, Osaka-Sayama 589-8511, Japan; Kindai University Hospital Global Research Alliance Center, Osaka-Sayama 589-8511, Japan

**Keywords:** extramammary Paget’s disease, gene expression profiling, human epidermal growth factor receptor 2, molecular targeted therapies

## Abstract

**Background:**

Extramammary Paget’s disease (EMPD) is a rare malignancy without established systemic therapy. EMPD shares molecular features with breast cancer, such as human epidermal growth factor receptor 2 (HER2) and hormone receptor (HR) expression, but their clinical relevance remains unclear.

**Materials and Methods:**

Tumors from 20 metastatic invasive EMPD cases were analyzed for molecular and biological features. Genomic features, transcriptomic profiles, and HER2 and HR expression status were investigated using immunohistochemistry, fluorescence in situ hybridization, and targeted-genome next-generation sequencing and nCounter BC360 panels. Metastatic breast cancer samples were used as a comparison to clarify metastatic EMPD’s clinical relevance.

**Results:**

Estrogen receptor expression was observed in 45% of EMPD tumors, while only 10% expressed progesterone receptor. HER2 was overexpressed in 30% of cases, and HER2-directed therapies were durably effective. Among 8 patients with NGS data, 63% (5/8) harbored oncogenic *ERBB2* alterations independent of HER2 expression. BC360 profiling revealed biological differences between EMPD and breast cancer, particularly poor biological compatibility for HR-positive tumors. Immune profiling showed that a subset of EMPD tumors exhibited CD8+ T-cell signatures and PD-1/PD-L1 gene expression comparable to triple-negative breast cancer. The median overall survival was 22.1 months (95% CI, 12.0-42.2), with 16 patients (80%) treated with systemic therapy, including anti-HER2 therapy, hormonal therapy, or cytotoxic therapies based on their molecular features.

**Conclusions:**

This study highlights the unique molecular and biological features of metastatic EMPD, emphasizing the need for tailored treatment approaches. This information should be used to guide future clinical strategies for metastatic EMPD.

Implications for Practice Metastatic extramammary Paget’s disease (EMPD) displays both overlapping and distinct biological features compared with breast cancer. Frequent *ERBB2* alterations, low progesterone receptor expression despite estrogen receptor positivity, and immune profiles resembling triple-negative breast cancer suggest the need for EMPD-specific strategies. Comprehensive molecular profiling may guide effective use of HER2-targeted agents and immune checkpoint inhibitors in select patients.

## Introduction

Extramammary Paget’s disease (EMPD) is a rare cutaneous ­cancer with the unique clinical feature of mostly occurring in genital or axillary areas. EMPD progresses slowly, and its prognosis is thus generally favorable, with local surgery usually being curative. Some patients, however, initially present with metastatic disease or develop systemic dissemination despite wide local excision, eventually resulting in a refractory or fatal clinical course. Despite this, studies of metastatic EMPD are lacking because of the rarity of this disease, and there are currently no well-established treatment strategies for metastatic EMPD.

Mammary Paget’s disease (MPD), in which Paget cells arise in the nipple or areolar regions of the breast, is treated as breast cancer because of their close anatomical locations. Treatment depends on the tumor’s hormone receptor (HR) and human epidermal growth factor receptor 2 (HER2) statuses. Classically, EMPD shares pathological features with MPD because Paget cells develop in extramammary areas. EMPD also shares some molecular features with breast cancer and MPD, with overexpression of HRs (estrogen receptor [ER] and progesterone receptor [PgR]) and/or HER2 (∼10% and ∼30% of patients with EMPD, respectively).^1–5^ Notably, several case reports indicated promising efficacy of antiestrogen therapy^6^ or anti-HER2 targeted therapies in EMPD,^7–10^ suggesting that breast cancer treatment strategies may also guide EMPD treatment; however, the rarity of the disease and consequent lack of a biological basis warrant comprehensive molecular and biological profiling of clinical EMPD to facilitate its appropriate management.

We retrospectively analyzed the treatment outcomes of patients with metastatic EMPD in relation to the clinical and pathological/molecular features, including HR and HER2 expression, along with systemic treatment outcomes for molecular-targeted therapies. We also compared clinical tumor samples between patients with EMPD and breast cancer, using comprehensive gene expression profiling (GEP). This comprehensive clinical and biological profiling provides an overall disease picture of EMPD, thus supporting the advanced care of patients with metastatic EMPD.

## Methods

### Patient characteristics and evaluation of treatment response

Patients with metastatic EMPD who visited our department between April 2007 and October 2019 were included in the study. The cutoff date for data collection was November 30, 2024. Patient characteristics and treatment, including next-generation sequencing (NGS) results, were reviewed retrospectively from medical records. Patients with metastatic breast cancer who visited our department between May 2017 and May 2019 were included for comparison. This study was conducted in accordance with the principles of the Declaration of Helsinki. The study protocol was approved by the institutional review board at Kindai University Faculty of Medicine (accession number, 31-001). Patient consent was obtained where appropriate, according to the study protocol.

### Immunohistochemistry

Formalin-fixed paraffin-embedded (FFPE) EMPD tumor tissues were sectioned (4 μm) and stained using the following primary antibodies, according to the respective manufacturers’ recommendations: HER2/*neu* (4B5, 790-2991, Roche, Basel, Switzerland), ER (6F11, ER-6F11-L-CE, Leica ­Biosystems, Buffalo Grove, IL), PgR (16, PGR-312-L-CE-S, Leica Biosystems), and Ki-67 (SP6, 418071, Nichirei Biosciences, Tokyo, Japan). Nuclear positivity for ER, PgR, and Ki-67 and membrane positivity for HER2 were calculated. HER2 immunostaining was scored based on the American Society of Clinical Oncology/College of American Pathologists Clinical Practice Guidelines HER2 staining algorithm for breast cancer.^11^ Clinicopathological immunohistochemi­stry (IHC) results for breast cancer patients were used for the analysis.

### Fluorescence in situ hybridization

*HER2* (or *ERBB2*) fluorescence in situ hybridization (FISH) analysis was performed using PathVysion HER-2 DNA Probe Kit (Abbott Molecular, Des Plaines, IL, USA) according to the manufacturer’s protocol. HER2/CEP17 (chromosome 17 probe) was counted in >20 tumor cells, and a HER2/CEP17 ratio ≥2.0 was considered amplified.

### Transcriptome analysis

Total RNA was isolated from 4-μm FFPE sections using an RNeasy FFPE Kit (Qiagen, Hilden, Germany) and assessed using a NanoDrop 2000 (Thermo Fisher Scientific, Waltham, MA, USA). ≥50 ng total RNA was hybridized to the NanoString Breast Cancer 360 Gene Expression Panel (NanoString Technologies, Seattle, WA, USA). Gene expression data were normalized to housekeeping genes, as per the manufacturer’s protocol, using nSolver 4.0 software (NanoString Technologies). Quality control was performed as described previously.^12^ PAM50 scores and gene signatures were provided with R version 3.3.2, as described previously.^13^^,^^14^

EMPD samples were compared with samples from patients with metastatic breast cancer who visited our department between May 2017 and May 2019. The samples were processed for GEP analysis in the same manner.

### Statistical analysis

This study was exploratory and hypothesis-generating in nature. Therefore, statistical tests were performed to identify potential associations rather than to formally confirm predefined hypothesis. No threshold of *P* was defined for statistical significance. Simple linear regression analysis was performed to assess the association between HER2 IHC score and ER positivity. Continuous variables were compared by the Wilcoxon rank sum test. Overall survival (OS), defined as the time from first diagnosis of metastatic disease to the date of death or last follow-up, was calculated by the Kaplan–Meier method. Log-rank test was used for survival comparison. All analyses were performed with GraphPad Prism version 8.0 (GraphPad Software, San Diego, CA, USA) or StataMP 16 (StataCorp LLC, College Station, TX, USA).

## Results

### Patients

We identified 22 patients with metastatic EMPD from medical records in our department. Two patients were excluded because no tissue specimens were available. The patient characteristics are shown in Table 1.

**Table 1.** Patient characteristics (*n* = 20).

Characteristics	No. of patients (%)
Median age (range), years	72 (48-87)
Sex	
Male	18 (90)
Female	2 (10)
Smoking history	
Former or current	9 (45)
Never	5 (25)
Unknown	6 (30)
ECOG-PS	
0	5 (25)
1	11 (55)
2	2 (10)
3	1 (5)
Unknown	1 (5)
Primary site	
Perineum	20 (100)
Disease status	
De-novo metastatic	9 (45)
Postoperative metastatic recurrence	11 (55)

Abbreviation: ECOG-PS, Eastern Cooperative Oncology Group performance status.

### IHC analysis

**Figure 1.**
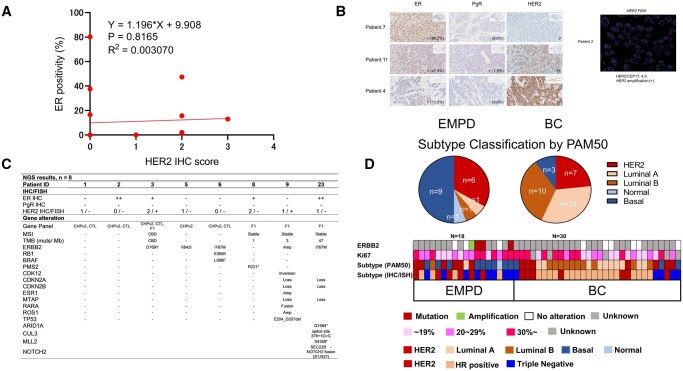
Molecular profiling in extramammary Paget’s disease. (A) Scatter plot of HER2 IHC score and ER positivity. (B) Representative IHC and FISH analyses of EMPD samples. (C) IHC, FISH and NGS results in patients whose NGS results were available (*n* = 8/20). (D) PAM50 classification and IHC and FISH results. ­Two EMPD (patients 19 and 23) and one breast cancer sample were excluded from the analysis because of low-quality mRNA. Abbreviations: EMPD, extramammary Paget’s disease; IHC, immunohistochemistry; FISH, fluorescence *in situ* hybridization; HER2, human epidermal growth factor receptor 2; ER, estrogen receptor; PgR, progesterone receptor; NGS, next-generation sequencing; BC, breast cancer; CHPv2, Ion Ampliseq cancer hotspot panel v2; CTL, Archer FusionPlex CTL panel; F1, FoundationOne CDx; MSI, microsatellite instability; TMB, tumor mutational burden; CBD, cannot be determined; amp, amplification; del, deletion; ++, ≥10%; +, ≥1%, <10%; -, <1%.

ER, PgR, HER2, and Ki-67 immunostaining and *HER2* FISH analysis were performed. We defined HR-positivity as ER and/or PgR IHC staining >1% and HER2-positivity as IHC 3+ or FISH-positive (HER2/CEP17 ≥ 2.0). The IHC profiling results are shown in Table 2. There were 9 patients with ER-positive tumors (45%), including 7 (35%) strongly positive. PgR was only expressed in 2 patients (10%), both with weak expression, while ER was strongly co-expressed. HER2 was equivocally expressed (2+) or overexpressed (3+) in the tumors from 6 (30%) patients, whereas all *HER2* FISH results were positive in those tumors. There was no association between ER and HER2 expression (Figure 1A). The median Ki-67of EMPD was 25.5 (range, 1.7-48.1), which was comparable to the results for breast cancer (median, 24; range, 2.8-53).

**Table 2.** IHC/FISH analysis (*n* = 20).

	No. of patients (%)
Molecular characteristic	
ER positive	9 (45)
1%-10%	2 (10)
>10%	7 (35)
PgR positive	2 (10)
1%-10%	2 (10)
>10%	0 (0)
HER2 positive^a^	6 (30)
IHC 3+	1 (5)
FISH positive	6 (30)
Ki-67	
<20%	7 (35)
≥20%	13 (65)
**Subtype classification by IHC/FISH**	
HR positive/HER2 negative	5 (25)
HER2 positive	6 (30)
HR negative/HER2 negative	9 (45)

Abbreviations: ER, estrogen receptor; FISH, fluorescence *in situ* hybridization; HER2, human epidermal growth factor receptor 2; HR, hormone receptor; IHC, immunohistochemistry; PgR, progesterone receptor.

a HER2 positive defined as HER2 IHC 3+ or *HER2* FISH positive.

Patients were classified into 3 subgroups: HR-positive/HER2-negative (5/20, 25%), HER2-positive (6/20, 30%), and HR-negative/HER2-negative (9/20, 45%), according to breast cancer subtype classification (Table 2). Representative results are shown in Figure 1B. The IHC and FISH results for breast cancer tissues (positive control) are shown in Table S1. These comprehensive HR/HER2 profiles highlight the potential of hormonal or anti-HER2 therapy for metastatic EMPD.

### Next-generation sequencing

NGS results were available for 8 of the 20 patients (Figure 1C). Five of the 8 showed *ERBB2* gene alterations (I767M [2 cases], D769Y and V842I, or HER2 amplification), all located in the kinase domain and all considered oncogenic, according to OncoKB (https://www.oncokb.org/). Two cases with *ERBB2* oncogenes also harbored a loss of genes on chromosome 9p21, including *CDKN2A*, *CDKN2B*, and *MTAP*, consistent with previous reports of EMPD.^15^^,^^16^ There was no microsatellite instability in any cases and no other oncogenic abnormalities, suggesting that *ERBB2* acts as a key driver gene in metastatic EMPD. Notably, these *ERBB2* oncogenes were not translated to HER2 protein overexpression, reminiscent of *ERBB2*-mutant metastatic non-small cell lung cancer, where the oncogenicity of *ERBB2* mutations do not require HER2 overexpression.^17^ In addition, these *ERBB2* mutations are infrequent (<5%) in breast cancer, suggesting a unique molecular feature of EMPD.^18^

### Transcriptome analysis

The above IHC and NGS results indicated shared molecular features between EMPD and breast cancer (overexpression of HR and HER2 proteins), as well as distinctive molecular features of metastatic EMPD (recurrent *ERBB2* oncogenes despite low HER2 protein expression). To clarify the biological profile of EMPD, we compared transcriptomic phenotypes of tumors from patients with EMPD and breast cancer, respectively. Twenty EMPD and 31 breast cancer specimens were subjected to nCounter BC360. Two EMPD and one breast cancer sample were excluded from the analysis because of low-quality mRNA (mRNA content normalization factor >10, following previous methods^12^).

PAM50 scores were derived from GEP, and EMPD and breast cancer samples were classified based on the PAM50 intrinsic breast cancer subtype (Figure 1D).^13^ PAM50 phenotypes were concordant with the IHC/FISH-based clinical phenotypes in breast cancer but not in EMPD. The discordance between the IHC and PAM50 subtypes for EMPD was ­particularly notable for the IHC-based HR type, in which 50% of IHC-­based HR-positive samples were considered as the HER2 subtype based on PAM50. Consistent with the discordance between the IHC and NGS profiles (Figure 1D), activating *ERBB2* mutations were not translated to the HER2 subtype, even based on PAM50. These discordances further indicated a unique biological feature of EMPD.

**Figure 2.**
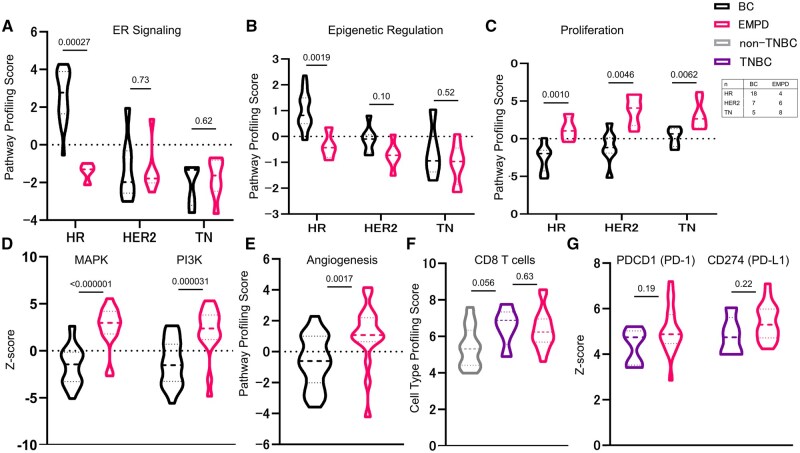
Transcriptome analysis of extramammary Paget’s disease and breast cancer samples. ER signaling (A), epigenetic regulation (B), and proliferation (C) pathway signatures in each IHC/FISH-based subtype were compared between BC (*n* = 30) and EMPD samples (*n* = 18). MAPK and PI3K pathway (D) and angiogenesis (E) signatures in BC and EMPD samples. CD8 T cell signatures (F) were compared between non-TNBC (*n* = 25), TNBC (*n* = 5), and EMPD samples. *PDCD1* and *CD274* gene expression were compared between TNBC and EMPD samples (G). Abbreviations: ER, estrogen receptor; IHC, immunohistochemistry; FISH, fluorescence *in situ* hybridization; HR, hormone receptor; HR, HR-positive/HER2-negative; HER2, HER2-positive; TN, triple negative, EMPD, extramammary Paget’s disease; BC, breast cancer; MAPK, mitogen-activated protein kinase; PI3K, phosphoinositide 3-kinase.

We further investigated the unique biological features of EMPD by comparing individual pathway signatures between EMPD and breast cancer samples. ER signaling was significantly higher in IHC-based HR-subtype breast cancer samples (Figure 2A) but was low in IHC-based HR-subtype EMPD, likely explaining the discrepancy between the IHC-based and PAM50-based classification for HR in EMPD (Figure 2A). Accordingly, the epigenetic regulation signature, as another hallmark of HR-positive breast cancer, was not expressed in IHC-HR-positive EMPD tumors (Figure 2B). Proliferation signaling was upregulated more in EMPD tumors than in breast cancer samples, regardless of IHC/FISH status (Figure 2C). In line with this, mitogen-activated protein kinase and phosphoinositide 3-kinase signatures, as key upstream signaling factors for cell cycling, were higher in EMPD than in breast cancer samples, regardless of IHC/FISH status, highlighting an aggressive feature of metastatic EMPD and suggesting that targeting these pathways may be a potential therapeutic target (Figure 2D). The angiogenesis signature, as an established therapeutic target for HER2-negative breast cancer,^19^ was further upregulated in EMPD tumors, supporting the use of anti-­angiogenesis therapies in this disease (Figure 2E). All other signatures are shown in Figures S1-S3 (see online supplementary material for a color version of this figure). Anti-programmed cell death protein 1 (PD-1) therapies are effective for triple-­negative breast cancer (TNBC), and we therefore carried out immune profiling of EMPD. The CD8 T cell signature was upregulated in TNBC compared with other types of breast cancer (*P* = .046, Figure 2F). Notably, some cases of EMPD had immune profiles (CD8 T cell signature: *PDCD1* [PD-1] mRNA, *CD274* [PD-ligand 1, PD-L1] mRNA) equivalent to TNBC, suggesting that anti-PD-1 therapies may be effective in some patients with EMPD (Figure 2F, G).

### Treatment outcome according to clinical and biological profiles

**Figure 3.**
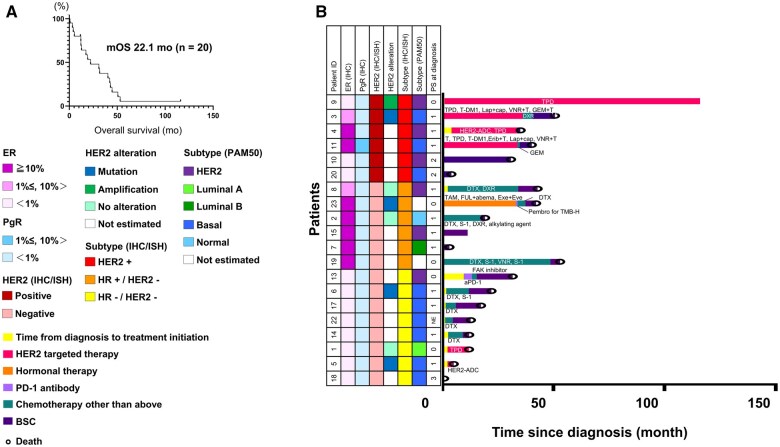
Overall survival according to molecular subtype and treatment. (A) Kaplan–Meier survival curve in patients with EMPD (*n* = 20). The median OS was 22.1 months (95% CI, 12.0-42.2 months). (B) Swimmer plot for individual patients, with molecular characteristics. Each horizontal bar represents the clinical course of a patient from diagnosis until death or last follow-up. The timing and type of systemic therapies are indicated by color codes as follows: pink, HER2-targeted therapies: orange, hormonal therapies: light purple, PD-1 antibody: green, chemotherapy not classified as above, purple: best supportive care (BSC). The yellow segment at the beginning of each bar denotes the time from diagnosis to the initiation of systemic therapy. Abbreviations: EMPD, extramammary Paget’s disease; mOS, median overall survival; ER, estrogen receptor; HR, hormone receptor; HER2, human epidermal growth factor receptor 2; IHC, immunohistochemistry; FISH; fluorescence *in situ* hybridization; PgR, progesterone receptor; PD-1, programmed cell death protein 1; PS, ECOG performance status; TPD, trastuzumab + pertuzumab + docetaxel; T-DM1, trastuzumab emtansine; Lap, lapatinib; Cap, capecitabine; VNR, vinorelbine; T, trastuzumab; GEM, gemcitabine; ADC, antibody-drug conjugate; Erib, eribulin; DTX, docetaxel; DXR, doxorubicin; TAM, tamoxifen; FUL, fulvestrant; abeam, abemaciclib; Exe, exemestane; Eve, everolimus; Pembro, pembrolizumab; TMB-H, tumor-mutational burden high; aPD-1, anti PD-1 antibody; FAK, focal adhesion kinase.

Anti-cancer treatments in patients with EMPD are summarized in Table 3 and Figure S4 (see online supplementary material for a color version of this figure). Most patients were treated for MPD, according to Breast Cancer Treatment Guidelines. Fifteen patients (75%) received systemic therapy, and 5 (25%) received best supportive care (BSC) alone. The median number of lines of systemic therapy was one (range, 0-7). One patient received tamoxifen, as previously reported,^6^ and 4 (including one previously reported patient^7^) received HER2-directed therapies, such as trastuzumab, pertuzumab, T-DM1, or lapatinib. Eleven patients received non-molecular-targeted cytotoxic therapy, and 5 patients received BSC alone. The median OS in all patients was 22.1 months (95% CI: 12.0-42.2; Figure 3A). The detailed treatment outcomes are summarized in Figure 3B. Notably, long-term survival for at least 2.9-9.6 years was observed in most patients with HER2-positive tumors treated with HER2-directed therapy-based regimens, but a durable therapeutic benefit was only observed in a small fraction of patients who were only suitable for conventional cytotoxic therapies. Anti-PD-1 therapy was not effective in 2 patients, including one with a high tumor mutational burden.

**Table 3.** Overall survival (*n* = 20).

Subtype	Median OS (95% CI)	*P*-value
HR+/HER2− (*n* = 5)	42.2 (3.2-NE)	
HER2+ (*n* = 6)	37.8 (4.1-NE)	
HR−/HER2− (*n* = 9)	12.8 (0.8-31.7)	
Treatment	Median OS (95% CI)	*P*-value
Log-rank test	-	.2666
Systemic therapy (*n* = 15)	31.7 (12.1-42.9)	
BSC alone (*n* = 5)	4.1 (0.8-NE)	
Log-rank test	-	.0131
Treatment of HER2+ EMPD	Median OS (95% CI)	*P*-value
HER2 targeted therapy (*n* = 4)	68.9 (35.2-NE)	
BSC alone (*n* = 2)	17.5 (4.1-NE)	
Log-rank test	-	.0177

Abbreviations: BSC, best supportive care; EMPD, extramammary Paget’s disease; HER2, human epidermal growth factor receptor 2; HR, hormone receptor; NE, not estimated; OS, overall survival.

## Discussion

Clinical decisions regarding treatment for metastatic disease of rare cancers are always challenging, given the lack of clinical data on the therapeutic outcomes of systemic therapies. Rare diseases preclude the option of large prospective clinical trials, leading to the need to rely on anecdotal evidence. Encouragingly, however, recent advances in precision therapy based on molecular features may overcome this clinical challenge, and some tumor-agnostic strategies have led to the development of new standard-of-care therapies, even for rare cancers, based on shared molecular phenotypes (eg, neurotrophic tyrosine receptor kinase-fusion-positive cancers, mismatch repair-deficient cancers).^20^^,^^21^ Even the efficacies of molecular-targeted therapies, however, may differ among tumor types, such as reduced efficacy of BRAF- or KRAS-targeted therapy in colon cancer, possibly because of differences in the biology underlying the respective tumor development. Detailed phenotyping is therefore particularly important for rare cancers, to facilitate the use of accurate and individualized precision therapy. Given the lack of clinical and translational evidence for metastatic EMPD to date, the results will support oncologists in providing clinical decisions. The results revealed that metastatic EMPD expectedly shares some features with metastatic breast cancer but also has distinctive features.

Almost half of all metastatic EMPD tumors in the present study expressed HRs, in contrast to previous studies that identified only ∼10% of resected EMPD tumors as HR-positive. This may indicate that metastatic or aggressive EMPD tends to be HR-positive. Notably, however, PgR was absent or barely expressed in metastatic EMPD tumors in our report (10% of tumors were weakly positive, and the rest were negative), compared with metastatic breast cancer, which strongly expressed PgR in most HR-positive cases (eg, 70%-80%).^22^^,^^23^ Given that low PgR expression is a negative predictive factor for anti-­hormone therapy efficacy,^24^^,^^25^ the potential of hormonal therapy may thus be limited in metastatic EMPD. In addition, our GEP revealed that HR-positive metastatic EMPD differed from HR-positive metastatic breast cancer. In breast cancer, HER2-low (ie, HER2 1+ or 2+/FISH−) is positively correlated with ER expression^26^; however, no such relationship was observed in metastatic EMPD in the present study. The collective evidence for the unique features of HR-positive EMPD should be considered when making clinical decisions about the use of anti-hormone therapy in patients with metastatic EMPD. Nevertheless, as noted in our previous case report,^6^ a certain subset of patients may benefit from anti-hormone therapy, and further studies including factors other than receptor status are warranted to identify such individuals.

Our data regarding HER2 status in metastatic EMPD are encouraging, given the tangible efficacy of HER2-targeted therapies in HER2-overexpressing EMPD tumors. The frequency of HER2-overexpressing tumors in metastatic EMPD was comparable to that for metastatic breast cancer. Intriguingly, oncogenic *ERBB2* mutations were more frequent compared with metastatic breast cancer. Although the current study could not provide sufficient evidence for HER2-directed therapy in these *ERBB2*-mutant metastatic EMPD tumors, anti-HER2 antibody–­drug conjugates should be considered, given the recent success of trastuzumab deruxtecan in *ERBB2*-mutant metastatic non-small-cell lung cancer, despite low HER2 protein expression levels. These data support the need for more clinical/translational studies of metastatic EMPD based on HER2 status, in relation to both overexpression and oncogenic mutations.

Given the broad success of anti-PD-1 therapies across tumor types, its role in metastatic EMPD is of clinical interest. The pretreatment immune environment, including pretreatment intratumoral immune cell infiltration or PD-L1 expression on cancer cells or antigen-presenting cells, predicts the efficacy of anti-PD-1 therapy across tumor types. This, together with the current finding that a subset of EMPD tumors showed an upregulated CD8 T cell signature, *CD273* (PD-1 gene), and *CD274* (PD-L1 gene), suggests that anti-PD-1 therapy is worth considering. Pretreatment evaluation of CD8+ cell infiltr­ation or PD-L1 expression levels may help to guide clinical decisions.

This study was limited by its small sample size and retrospective nature, with no control treatment arms. Therefore, this study remains exploratory and hypothesis-generating. However, the rarity of this condition means that even such small retrospective cohort studies have previously been impossible. Thus, despite the small sample size, the present findings offer valuable insight into potential therapeutic targets and biological heterogeneity in metastatic EMPD.

In conclusion, this study was the first to interrogate the mole­cular and biological profiles of metastatic EMPD using clinical samples from a cohort of patients and to compare them with metastatic breast cancer to help determine the clinical features of metastatic EMPD. The results will facilitate the development of effective personalized medicine approaches for metastatic EMPD.

## Supplementary Material

oyaf235_Supplementary_Data

## Data Availability

All data generated in this study are accessible from the corresponding author upon reasonable inquiry.

## References

[1] de Leon ED , Carcangiu ML, Prieto VG, et al Extramammary Paget disease is characterized by the consistent lack of estrogen and progesterone receptors but frequently expresses androgen receptor. Am J Clin Pathol. 2000;113:572-575. 10761460 10.1309/P756-XXCB-TV71-U4XV

[2] Liegl B , Horn L-C, Moinfar F. Androgen receptors are frequently expressed in mammary and extramammary Paget’s disease. Mod Pathol. 2005;18:1283-1288. 15920545 10.1038/modpathol.3800437

[3] Tanaka R , et al Human epidermal growth factor receptor 2 protein overexpression and gene amplification in extramammary Paget disease. Br J Dermatol. 2013;168:1259-1266. 23360223 10.1111/bjd.12249

[4] Brummer O , Stegner HE, Bohmer G, Kuhnle H, Petry KU. HER-2/neu expression in Paget disease of the vulva and the female breast. Gynecol Oncol. 2004;95:336-340. 15491754 10.1016/j.ygyno.2004.07.043

[5] Richter CE , Hui P, Buza N, et al HER-2/NEU overexpression in vulvar Paget disease: the Yale experience. J Clin Pathol. 2010;63:544-547. 20418225 10.1136/jcp.2010.077446

[6] Isomoto K , Haratani K, Watanabe S, et al Successful treatment of a case of hormone receptor-positive metastatic extramammary Paget disease with tamoxifen. Invest New Drugs. 2022;40:194-197. 34463889 10.1007/s10637-021-01168-5

[7] Watanabe S , Takeda M, Takahama T, et al Successful human epidermal growth receptor 2-targeted therapy beyond disease progression for extramammary Paget’s disease. Invest New Drugs. 2016;34:394-396. 26856856 10.1007/s10637-016-0329-8

[8] Barth P , Al-Saleem ED, Edwards KW, et al Metastatic extramammary Paget’s disease of scrotum responds completely to single agent trastuzumab in a hemodialysis patient: case report, molecular profiling and brief review of the literature. Case Rep Oncol Med. 2015;2015:895151. 25692060 10.1155/2015/895151 PMC4322830

[9] Wakabayashi S , Togawa Y, Yoneyama K, et al Dramatic clinical response of relapsed metastatic extramammary Paget’s disease to trastuzumab monotherapy. Case Rep Dermatol Med. 2012;2012:401362. 23259081 10.1155/2012/401362 PMC3505941

[10] Takahagi S , Noda H, Kamegashira A, et al Metastatic extramammary Paget’s disease treated with paclitaxel and trastuzumab combination chemotherapy. J Dermatol. 2009;36:457-461. 19691751 10.1111/j.1346-8138.2009.00676.x

[11] Wolff AC , Hale Hammond ME, Allison KH, et al HER2 testing in breast cancer: American Society of Clinical Oncology/College of American Pathologists Clinical Practice Guideline focused update summary. J Oncol Pract. 2018;14:437-441. 29920138 10.1200/JOP.18.00206

[12] Haratani K , Nakamura A, Mamesaya N, et al Tumor microenvironment landscape of NSCLC reveals resistance mechanisms for programmed death-Ligand 1 blockade after chemoradiotherapy: a multicenter prospective biomarker study (WJOG11518L: SUBMARINE). J Thorac Oncol. 2023;18:1334-1350. 37364849 10.1016/j.jtho.2023.06.012

[13] Parker JS , Mullins M, Cheang MCU, et al Supervised risk predictor of breast cancer based on intrinsic subtypes. J Clini Oncol. 2009;27:1160-1167. 10.1200/JCO.2008.18.1370 PMC2667820 19204204

[14] Zhao X , Rødland EA, Tibshirani R, Plevritis S. Molecular subtyping for clinically defined breast cancer subgroups. Breast Cancer Res. 2015;17:29. 25849221 10.1186/s13058-015-0520-4 PMC4365540

[15] Ishida Y , Kakiuchi N, Yoshida K, et al Unbiased detection of driver mutations in extramammary Paget disease. Clin Cancer Res. 2021;27:1756-1765. 33323405 10.1158/1078-0432.CCR-20-3205

[16] Iwasawa O , Ikegami M, Miyagawa T, et al Association of genetic alterations with prognosis in extramammary Paget’s disease: insights into the involvement of somatic CDKN2A variants in poor prognosis. Br J Dermatol. 2024;192:46–54. 39172540 10.1093/bjd/ljae337

[17] Shih J-Y. ERBB2 amplification in NSCLC: how many faces? J Thorac Oncol. 2024;19:668-670. 38719422 10.1016/j.jtho.2024.02.001

[18] Robichaux JP , Elamin YY, Vijayan RSK, et al Pan-Cancer landscape and analysis of ERBB2 mutations identifies poziotinib as a clinically active inhibitor and enhancer of T-DM1 activity. Cancer Cell. 2019;36:444-457.e447. 31588020 10.1016/j.ccell.2019.09.001 PMC6944069

[19] Miller K , Wang M, Gralow J, et al Paclitaxel plus bevacizumab versus paclitaxel alone for metastatic breast cancer. N Engl J Med. 2007;357:2666-2676. 18160686 10.1056/NEJMoa072113

[20] Cocco E , Scaltriti M, Drilon A. NTRK fusion-positive cancers and TRK inhibitor therapy. Nat Rev Clin Oncol. 2018;15:731-747. 30333516 10.1038/s41571-018-0113-0 PMC6419506

[21] Marabelle A , Le DT, Ascierto PA, et al Efficacy of pembrolizumab in patients with noncolorectal high microsatellite instability/mismatch repair-deficient cancer: results from the phase II KEYNOTE-158 study. J Clin Oncol. 2020;38:1-10. 31682550 10.1200/JCO.19.02105 PMC8184060

[22] Colleoni M , Viale G, Zahrieh D, et al Expression of ER, PgR, HER1, HER2, and response: a study of preoperative chemotherapy. Ann Oncol. 2008;19:465-472. 17986623 10.1093/annonc/mdm509

[23] Kurozumi S , Matsumoto H, Hayashi Y, et al Power of PgR expression as a prognostic factor for ER-positive/HER2-negative breast cancer patients at intermediate risk classified by the Ki67 labeling index. BMC Cancer. 2017;17:354. 28532429 10.1186/s12885-017-3331-4 PMC5441075

[24] Dowsett M , Allred C, Knox J, et al Relationship between quantitative estrogen and progesterone receptor expression and human epidermal growth factor receptor 2 (HER-2) status with recurrence in the arimidex, tamoxifen, alone or in combination trial. J Clin Oncol. 2008;26:1059-1065. 18227529 10.1200/JCO.2007.12.9437

[25] Prat A , Cheang MCU, Martín M, et al Prognostic significance of progesterone receptor-positive tumor cells within immunohistochemically defined luminal a breast cancer. J Clin Oncol. 2013;31:203-209. 23233704 10.1200/JCO.2012.43.4134 PMC3532392

[26] Tarantino P , Jin Q, Tayob N, et al Prognostic and biologic significance of ERBB2-low expression in early-stage breast cancer. JAMA Oncol. 2022;8:1177-1183. 35737367 10.1001/jamaoncol.2022.2286 PMC9227690

