# Supplementary material for: Clinical and molecular landscape of metastatic extramammary Paget’s disease
Source: Oncologist. 2025 Jul 28;30(9):oyaf235. doi: 10.1093/oncolo/oyaf235 (PMC12422315; doi:10.1093/oncolo/oyaf235)
Supplement: oyaf235_Supplementary_Data [file oyaf235_supplementary_data.zip › EMPD_Suppl_Table_Watanabe.docx]

| **Table S1. IHC/FISH analysis of breast cancer samples (n = 31)** | |  |
| --- | --- | --- |
| **Molecular characteristic** | **No. of patients (%)** | |
| ER positive | 23 (74) | |
| 1%–10% | 0 (0) | |
| >10% | 23 (74) | |
| PgR positive | 22 (71) | |
| 1%–10% | 2 (6) | |
| >10% | 20 (65) | |
| HER2 positive^a^ | 7 (23) | |
| IHC 3+ | 7 (23) | |
| FISH positive | 0 (0) | |
| Ki-67 |  | |
| <20% | 9 (29) | |
| ≥20% | 16 (52) | |
| NE | 6 (19) | |
| **Subtype classification by IHC/FISH** | **No. of patients (%)** | |
| HR positive / HER2 negative | 19 (61) | |
| HER2 positive | 7 (23) | |
| HR negative / HER2 negative | 5 (16) | |
| Abbreviations: IHC, immunohistochemistyry; FISH, fluorescence *in situ* hybridization; ER, estrogen receptor; PgR, progesterone receptor; HER2, human epidermal growth factor receptor 2; HR, hormone receptor; NE, not estimated  ^a^HER2 positive defined as HER2 IHC 3+ or *HER2* FISH positive | |  |
